# Early beginnings - the emergence of complex signaling systems and cell-to-cell communication

**DOI:** 10.1186/1478-811X-8-16

**Published:** 2010-07-12

**Authors:** Stephan M Feller

**Affiliations:** 1 Cell Signalling Group, Weatherall Institute of Molecular Medicine, University of Oxford, Oxford OX3 9DS, UK

## 

The protein universe is currently still expanding [1] and so is the realm of cellular signaling proteins. Although in selected cases the genome size and number of genes in a species may substantially decrease as a reflection of a very narrow ecological niche and specialised lifestyle [2], overall there is an ever-increasing complexity in cellular signalling networks clearly evident throughout evolution.

For example, tyrosine kinases, phosphotyrosine-recognition domains (SH2 s etc.), and also the ancestral forms of large multi-docking proteins with folded N-termini and long 'intrinsically disordered' tails, like the p130Cas, Gab or IRS family proteins [3-5], which mediate high molecular weight signal transduction and integration complexes, emerged early during metazoan evolution [6]. Later on, genome duplications occurring during vertebrate evolution [7] allowed the emergence of small families of signaling proteins from single ancestor proteins. So compared to *Drosophila *and *C. elegans*, which typically have still one prototype protein, *Homo sapiens *has often three or four close relatives.

But why, when and how did signaling pathways and networks emerge in the first place? Clearly, the key to survival for all cells and organisms is to sense and respond appropriately to their natural surroundings. The sensing of the environment and signaling are very intimately linked, so it does not seem unlikely that a prokaryotic chemotaxis system [8] represents the first form of a cellular signaling system. The earliest presumed fossil records (stromatolites) seem to indicate that cellular life forms may have evolved over three billion years ago and it is hard to imagine that these early life forms would have been able to thrive for long without an environmental recognition and response system.

And when did multicellular organisms, which needed more complex signaling systems, including cell-to-cell communication, actually start to emerge? A new study now suggests a point in time over 2 billion years ago [9]. Macroscopically visible fossil records of up to a dozen centimetres in size found in equatorial Africa (southeastern Gabon), if interpreted correctly, point to the emergence of multicellularity relatively soon after the onset of the 'great oxidation event' [10]. These newly reported fossils form flat sheets with scalloped margins and noticeable radiating structures that seem to indicate coordinated growth. They seem to be unlike any known prokaryotic species, begging the question of whether they may actually represent very early eukaryotes.

Obviously we will be unable to query any ancient nucleic acids in this matter, but further analyses of these fossils may still eventually yield more insight into the very early days of cell - cell communication.

## References

[B1] Povolotskaya IS Kondrashov FA Sequence space and the ongoing expansion of the protein universe Nature 465 922 926 10.1038/nature09105 20485343

[B2] Kirkness EF Haas BJ Sun W Braig HR Perotti MA Clark JM Lee SH Robertson HM Kennedy RC Elhaik E Genome sequences of the human body louse and its primary endosymbiont provide insights into the permanent parasitic lifestyle Proc Natl Acad Sci USA in press 10.1073/pnas.1003379107 PMC2901460 20566863

[B3] Wohrle FU Daly RJ Brummer T Function, regulation and pathological roles of the Gab/DOS docking proteins Cell Commun Signal 2009 7 22 10.1186/1478-811X-7-22 19737390 PMC2747914

[B4] Mardilovich K Pankratz SL Shaw LM Expression and function of the insulin receptor substrate proteins in cancer Cell Commun Signal 2009 7 14 10.1186/1478-811X-7-14 19534786 PMC2709114

[B5] Tikhmyanova N Little JL Golemis EA CAS proteins in normal and pathological cell growth control Cell Mol Life Sci 67 1025 1048 10.1007/s00018-009-0213-1 19937461 PMC2836406

[B6] Harkiolaki M Tsirka T Lewitzky M Simister PC Joshi D Bird LE Jones EY O'Reilly N Feller SM Distinct binding modes of two epitopes in Gab2 that interact with the SH3C domain of Grb2 Structure 2009 17 809 822 10.1016/j.str.2009.03.017 19523899

[B7] Van de Peer Y Maere S Meyer A The evolutionary significance of ancient genome duplications Nat Rev Genet 2009 10 725 732 10.1038/nrg2600 19652647

[B8] Wuichet K Zhulin IB Origins and diversification of a complex signal transduction system in prokaryotes Sci Signal 3 ra50 10.1126/scisignal.2000724 20587806 PMC3401578

[B9] El Albani A Bengtson S Canfield DE Bekker A Macchiarelli R Mazurier A Hammarlund EU Boulvais P Dupuy JJ Fontaine C Fürsich FT Gauthier-Lafaye F Janvier P Javaux E Ossa Ossa F Pierson-Wickmann A-C Riboulleau A Sardini P Vachard D Whitehouse M Meunier A Large colonial organisms with coordinated growth in oxgenated environments 2.1 Gyr ago Nature 2010 466 100 104 10.1038/nature09166 20596019

[B10] Sessions AL Doughty DM Welander PV Summons RE Newman DK The continuing puzzle of the great oxidation event Curr Biol 2009 19 R567 574 10.1016/j.cub.2009.05.054 19640495

